# A Systematic Review on Artificial Intelligence Evaluating Metastatic Prostatic Cancer and Lymph Nodes on PSMA PET Scans

**DOI:** 10.3390/cancers16030486

**Published:** 2024-01-23

**Authors:** Jianliang Liu, Thomas P. Cundy, Dixon T. S. Woon, Nathan Lawrentschuk

**Affiliations:** 1 E.J. Whitten Prostate Cancer Research Centre, Epworth Healthcare, Melbourne, VIC 3005, Australia; jianliangl1@student.unimelb.edu.au (J.L.);; 2 Department of Urology, The Royal Melbourne Hospital, University of Melbourne, Melbourne, VIC 3052, Australia; 3 Department of Surgery, University of Melbourne, Melbourne, VIC 3052, Australia; 4 Discipline of Surgery, University of Adelaide, Adelaide, SA 5005, Australia

**Keywords:** artificial intelligence, convolutional neural network, deep learning, machine learning, prostate cancer, PSMA PET

## Abstract

**Simple Summary:**

This systematic review demonstrated that artificial intelligence (AI) can help detect metastatic prostate cancer with or without lymph node involvement on prostate-specific membrane antigen (PSMA) PET scans with high accuracy. Additional benefits of AI include the ability to estimate the volume of metastatic cancer, prognosticate, and differentiate bony metastasis from post-radiotherapy bone changes. AI can also improve workflow by helping to standardize reporting and automate time-consuming tasks. However, given the variable sensitivity and positive predictive value of AI, it is recommended that an experienced nuclear medicine physician proofread the final report. Larger studies producing more consistent results are needed before AI can be fully integrated into PSMA reporting.

**Abstract:**

Early detection of metastatic prostate cancer (mPCa) is crucial. Whilst the prostate-specific membrane antigen (PSMA) PET scan has high diagnostic accuracy, it suffers from inter-reader variability, and the time-consuming reporting process. This systematic review was registered on PROSPERO (ID CRD42023456044) and aims to evaluate AI’s ability to enhance reporting, diagnostics, and predictive capabilities for mPCa on PSMA PET scans. Inclusion criteria covered studies using AI to evaluate mPCa on PSMA PET, excluding non-PSMA tracers. A search was conducted on Medline, Embase, and Scopus from inception to July 2023. After screening 249 studies, 11 remained eligible for inclusion. Due to the heterogeneity of studies, meta-analysis was precluded. The prediction model risk of bias assessment tool (PROBAST) indicated a low overall risk of bias in ten studies, though only one incorporated clinical parameters (such as age, and Gleason score). AI demonstrated a high accuracy (98%) in identifying lymph node involvement and metastatic disease, albeit with sensitivity variation (62–97%). Advantages included distinguishing bone lesions, estimating tumour burden, predicting treatment response, and automating tasks accurately. In conclusion, AI showcases promising capabilities in enhancing the diagnostic potential of PSMA PET scans for mPCa, addressing current limitations in efficiency and variability.

## 1. Introduction

Prostate cancer (PCa) represents one of the leading causes of cancer-related mortality [1,2,3]. At diagnosis, 13% of PCa patients will have regional lymph node involvement, and 8% will have distant metastasis [1]. The most common site of metastatic PCa (mPCa) involvement is the bone, accounting for up to 90% of mPCa. Visceral organ involvement, such as in the lung, liver, adrenal, and brain, is less common [4]. When compared to localized PCa, the 5-year survival rate of mPCa declines significantly from 100% to 34.1% [1]. Early detection of mPCa is crucial for treatment institutions. Previous Cochrane reviews have demonstrated that early administration of androgen deprivation therapy (ADT) in mPCa improves the time to death from any cause of mortality, and decreases the rate of skeletal fractures [5].

Prostate-specific membrane antigen (PSMA) is a transmembrane glycoprotein that is upregulated in PCa [6]. The use of radiotracers with an affinity to PSMA in whole-body PET scans (PSMA PET) enables the detection of mPCa with high diagnostic accuracy. Currently, two PSMA tracers have received U.S. Food and Drug Administration (FDA) approval: Gallium 68 PSMA-11 (Ga 68 PSMA-11) and Pylarify (piflufolastat F 18) [7,8]. Conventional staging scans of PCa involve a computerized tomography of the abdomen and pelvis (CT AP) combined with a whole-body bone scan (WBBS). However, the ProPSMA trial showcased the superiority of PSMA PET-CT which has since displaced conventional staging scans [9]. A recent meta-analysis further cements the excellent diagnostic performance of PSMA PET for lymph nodes and bony metastasis, with the area under curves (AUC) of 0.95 and 0.99 respectively [10]. However, similar to other forms of medical imaging, reporting of PSMA PET is susceptible to inter-reader variability [11]. The efforts to standardize reporting with tools such as the prostate cancer molecular imaging standardized evaluation (PROMISE) criteria, the European Association of Nuclear Medicine (EANM) criteria, and the PSMA reporting and data system (PSMA-RADS) have improved inter-reader reproducibility [12]. Nevertheless, these tools can be labour-intensive and time-consuming.

There is considerable interest in integrating artificial intelligence (AI) into medical imaging given its ability to automate and its potential to leverage radiomics, which may be imperceptible to the naked eye. These complex AI algorithms have demonstrated improved diagnostic accuracy for staging in colorectal and lung cancer [13,14]. In prostate cancer, the automated PROMISE (aPROMISE) deep learning (DL) software developed by EXINI Diagnostics AB based on the PROMISE criteria has gained FDA approval [15,16]. The aPROMISE software first analyses the CT component of the PSMA PET-CT to automatically segment it into anatomical regions. Subsequently, the PSMA PET image is analyzed to detect metastasis. aPROMISE then merges the anatomical information and quantifies the tracer uptake to generate the miPSMA score. The miPSMA score was initially proposed in the original PROMISE criteria to assist in standardized reporting of PSMA expression in relation to blood pool, parotid gland, liver, or spleen [15]. Therefore, by leveraging DL, aPROMISE automates the labour-intensive task of anatomical segmentation and PSMA uptake quantification.

Belal et al.’s literature review has provided an excellent overview of the various applicability of AI in PSMA PET scans [17]. However, there is a lack of systematic review providing an in-depth analysis of how AI can be used on PSMA PET scans for PCa staging. This systematic review aims to evaluate the current role of AI in evaluating PSMA PET scans for PCa with distal metastasis and/or lymph node involvement.

## 2. Materials and Methods

### 2.1. Literature Search Strategy

This systematic review was registered on PROSPERO (international prospective register of systematic reviews) under the ID CRD42023456044. The preferred reporting items for systematic reviews and meta-analyses (PRISMA) guidelines were used (see Supplementary Materials). A comprehensive literature search was performed on Medline, Embase, and Scopus. Key search terms used include artificial intelligence, machine learning, deep learning, prostate cancer, and PSMA PET.

### 2.2. Eligibility Criteria

The population, intervention, comparator, and outcome (PICO) criteria were used to guide this systematic review. The population included all PCa patients who underwent a PSMA PET scan for staging of lymph node and distal metastasis. The intervention in question is the use of AI to evaluate PSMA PET. This includes machine learning (ML) which is a subset of AI. ML consists of complex algorithms which learn from experience (data) to recognise patterns and make predictions [18]. These data could be provided in the early development stage as training data, or later in the development after the training phase as validation data to fine-tune the algorithm. Testing data is the final data set used to evaluate the algorithm’s performance. DL is a subset of ML which uses many layers of the network to mimic the brain’s neuron network to learn and make decisions [19]. Convolutional neural networks (CNN) are a specific type of DL which processes visual data [20].

The comparator will either be against benign lesions or a nuclear physician report. The primary endpoint of this systematic review was to evaluate the ability of AI to improve the reporting of metastatic PCa (mPCa) or lymph node involvement on PSMA PET scans. Areas of PSMA PET scan reporting of interest include diagnostic accuracy, sensitivity, ability to differentiate from benign lesions, and standardization of reporting. The definition of metastasis and lymph node (regional versus non-regional) was according to the Tumour, Node, Metastasis (TNM) classification by the Union for International Cancer Control (UICC) 8th edition, 2017 [21]. The secondary endpoint of this study was to assess if AI could assess metastatic disease on PSMA PET-CT for prognosis or treatment response. All English language original articles published from inception to July 2023 were considered. The following types of studies were excluded: studies utilising only non-PSMA based radiotracers, studies utilising only the CT component of PSMA PET-CT without incorporating PET component, studies evaluating intra-prostatic lesions only, case reports, reviews, letters to journals, and conference abstracts.

### 2.3. Screening and Study Selection

Titles, abstracts, and full-text screening were performed independently by two authors (J.L and T.C) and any unresolved conflicts were resolved by the senior author. Relevant articles found in citations of included articles but not during the initial search will be included if eligibility criteria are met. The only automation tool used was Covidence to assist in the screening process and removal of duplicated articles. No artificial intelligence tools or software were used in the writing of this systematic review.

### 2.4. Quality and Risk of Bias Assessment

The standardized reporting of machine learning applications in urology (STREAM-URO) 26-item checklist was used to assess the quality of each article [22]. The STREAM-URO framework was created to ensure the quality of studies published, improve reproducibility and interpretation of results, and increase engagement and literacy of machine learning within the urological community. The prediction model risk of bias assessment tool (PROBAST) was used to assess the risk of bias (ROB) and the applicability of diagnostic and prognostic prediction model studies [23].

## 3. Results

### 3.1. Screening Process

**Figure 1:**
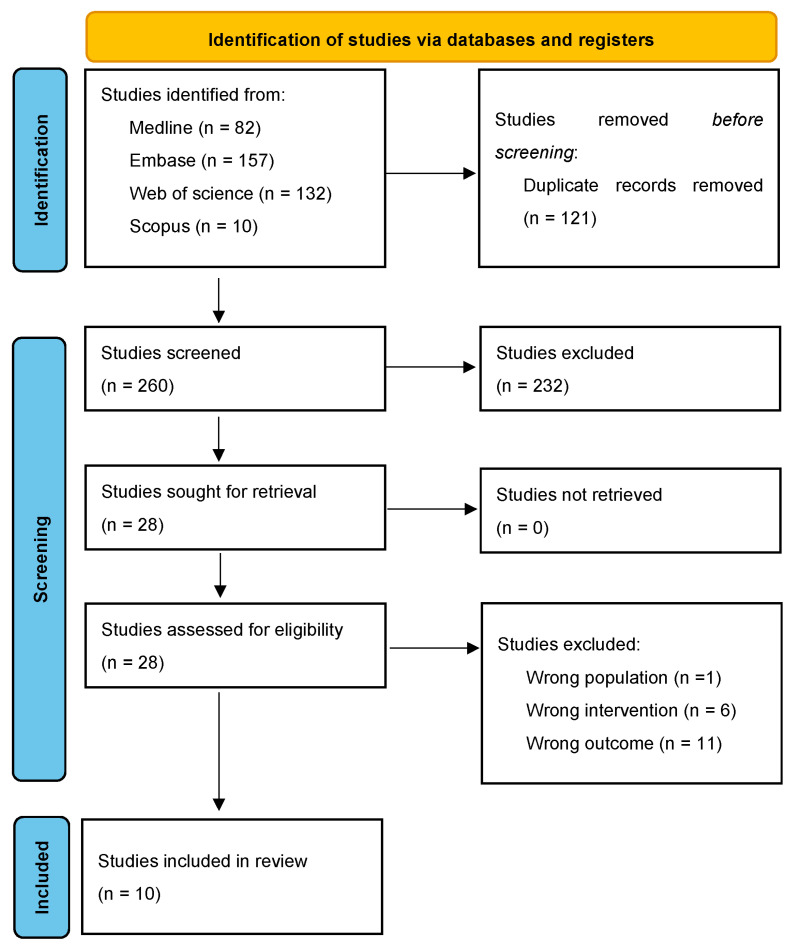
PRISMA 2020 flow diagram.

The search yielded 249 articles, 80 of which were duplicates (see Figure 1). After the exclusion of 141 articles during the title and abstract screening, 28 studies remained for full-text review. Eighteen studies were excluded during the full-text review due to insufficient sample size (*n* = 1), wrong intervention (*n* = 4), and wrong outcome (*n* = 13). During the full-text review, one additional eligible study by Nickols et al. [24] was found from the citations of the included studies. This study was absent from the original search most likely due to the absence of any AI-related medical subject headings (MeSH) terms in its title and abstract.

### 3.2. Characteristics of Included Studies

Of the 11 included studies, only one study by Kendrick et al. [25] was prospective, the remaining 10 were retrospective in nature (see Table 1). Tracers being used were ^18^F-PSMA (*n* = 5) and ^68^Ga-PSMA (*n* = 6). The types of AI algorithms used were as follows: ML (*n* = 5), DL (*n* = 2), and CNN (*n* = 4). The study by Moazemi et al. [26] was the only AI model which incorporated clinical parameters (such as age, Gleason score, and prostate-specific antigen (PSA)). The remaining 10 studies used AI models developed using radiological parameters only.

The objectives of the included studies were to assess the ability of AI to do the following: reduce inter-reader variability (*n* = 2), detect suspicious lesions only (*n* = 3), detect suspicious lesions and classify them anatomically (*n* = 1), detect and quantify tumour burden (*n* = 1), differentiate bony metastasis from sclerotic bone lesion which has completely responded to treatment (*n* = 1), predict treatment response to 177Lu-PSMA (*n* = 1), quantify treatment response of metastatic disease and correlate to PSA (*n* = 1), extract prognostic biomarkers (*n* = 1).

### 3.3. Quality and Risk of Bias Assessment of Included Studies

**Figure 2:**
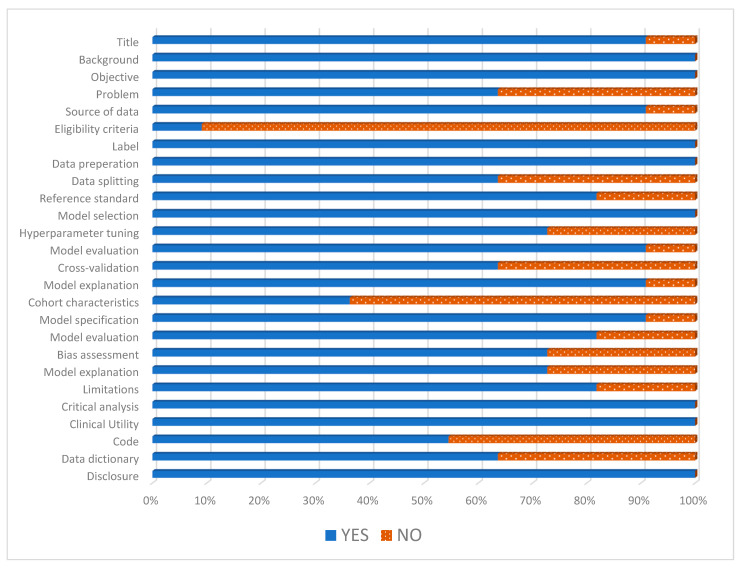
Standardized reporting of machine learning applications in urology (STREAM-URO) grading of the included studies.

The mean STREAM-URO score of the 11 studies was 21 out of 28 (see Figure 2). The main areas where studies scored the least were cohort characteristic (*n* = 4) as only four studies described the age and PSA of the included patients [24,25,26,33] and eligibility criteria (*n* = 1) as only one of the included studies described their exclusion criteria [33].

PROBAST assessment showed low overall ROB in ten studies and low overall applicability concerns in seven studies (see Table 2). One study had both high concerns for overall ROB and applicability as they did not use any separate dataset for validation or testing [33]. Another study had high concerns about applicability due to the inclusion of both PSMA PET and FDG PET scans in their training data [30]. The third study had unclear applicability as it aimed to evaluate if AI reduces inter-reader variability, but they did not compare their finding with inter-reader variability without AI [24]. Another study had unclear applicability as it was evaluating treatment response but included a heterogeneous group of patients being treated with surgery, radiotherapy, or ADT [34].

### 3.4. AI’s Ability to Standardise Staging of PCa on PSMA PET Scans

aPROMISE was used in three of the studies. Nickols et al. [24] demonstrated aPROMISE assisted reading resulted in low inter-reader variability between two nuclear medicine physicians in terms of PCa staging. Cohen pairwise k-agreement between the two nuclear medicine physicians were 0.82 for miN0M0, 0.90 for miN1M0 (presence of regional lymph node disease only), 0.77 for miN0M1b (presence of bony metastatic disease only). The study by Johnnson et al. [27], also demonstrated the high accuracy of segmentation by aPROMSIE when compared to manual segmentation by experienced nuclear medicine physicians. The Dice scores were as follows: bone segmentations (ranging from 0.88 to 0.95), thoracic aorta (0.89), and liver (0.97). Capobianco et al. [30] developed a CNN independent of aPROMISE which demonstrated an accuracy of 77% (CI: 70.0–83.4) for anatomical location classification of suspicious findings.

Leung et al. [28] developed a DL algorithm based on radiomic and anatomical tissue type information to categorise lesions into PSMA-RADS scores. PSMA-RADS is another tool used to standardise reporting of PSMA PET scans by utilising anatomy and PSMA avidity to classify lesions into a five-point scale which reflects the likelihood of the presence of PCa [35]. The DL by Leung et al. could assign a PSMA-RADS score at a patient level with a relatively high area under the receiver operating characteristic curve (AUROC) of 0.9, and an accuracy of 0.77. The findings were not as accurate at a lesion level with an AUROC of 0.87, and accuracy of 0.52. PSMA-RADS were further subdivided into three groups Group 1 (PSMA-RADS-1 and -2), Group 2 (PSMA-RADS-3), Group 3 (PSMA-RADS-4 and -5) and their mean probability scores were 0.19, 0.75, 0.86, respectively.

### 3.5. AI’s Role in Diagnosing Metastasis Disease on PSMA Pet Scans

In terms of detecting bony metastasis by DL, aPROMISE had 86.7% sensitivity [27], and another DL algorithm developed by Trägårdh et al. [29] only had a 62% sensitivity. Although the sensitivity of the DL algorithm by Trägårdh et al. was lower than aPROMISE, in its own analysis, the DL algorithm had a higher sensitivity of detecting bony metastasis as compared to the nuclear medicine physician (62% versus 59%). However, the DL by Trägårdh et al. does have a lower positive predicting value (PPV) of 40.5% as compared to the nuclear medicine physician of 58.7%.

Two of the included studies described the ability of their ML to detect all types of suspicious uptake outside of the prostate without differentiating between non-regional lymph nodes or non-lymphatic distal metastasis. The area under the curve (AUC), sensitivity, and specificity by Erle et al. [31] were 0.98, 97%, and 83%, and the study by Moazemi et al. [32] was 0.98, 94%, and 89%, respectively. The study by Capobianco et al. [30] developed a CNN to detect any suspicious uptake on ^68^Ga-PSMA PET-CT found to have an average precision of 80.4% [CI: 71.1–87.8], sensitivity of 81.1%, and positive predicting value of 66.8%.

### 3.6. AI’s Role in Diagnosing Lymph Node Involvement on PSMA PET Scans

Only two studies designed their AI to identify regional lymph nodes. Firstly, it was Capobianco et al.’s [30] CNN which demonstrated an 81% agreement for identified regional lymph nodes when compared to the expert reviewer. The study by Johnsson et al. [27] demonstrated that aPROMISE was able to identify suspicious regional lymph nodes with a 91.5% sensitivity. In a separate analysis by Johnsson et al. aPROMISE had a 90.6% sensitivity for detecting all types of lymph nodes (both regional and non-regional). The DL by Trägårdh et al., 2023 could identify all types of lymph nodes with a sensitivity of 79.1%, and PPV of 39.2% (as compared to 77.9% sensitivity and 78.3% PPV by nuclear medicine physician).

### 3.7. Estimating Tumour Burden and Prognosis

In addition to detecting lymph node, and bony metastasis, the DL developed by Trägårdh et al. [29] could also detect local or intraprostatic PCa recurrence. This is outside the scope of this systematic reference, but the sensitivity of detecting prostate/local recurrence was 78.7%. Subsequently, the DL was taught to measure markers of tumour burden which included total lesion volume (TLV) and total lesion uptake (TLU). TLV was the combination of the volume of all positive voxels. The TLU was first calculated for each lesion by dividing the mean standardized uptake values (SUVmean) by the TLV. The total TLU is the summation of all the TLU per lesion in each patient. The estimated tumour burden by the DL was very similar to the three nuclear medicine physicians’ calculation with statistical significance on the Spearman rank correlation test (ranging from R = 0.53 to R = 0.83).

Kendrick et al. [25] also developed a CNN to predict TLV and TLU but the CNN was trained on scans of patients with biochemically recurrent (BCR) PCa after definitive treatment. At the patient level, the accuracy, sensitivity, specificity, and PPV were 94.5%, 93.3%, 96.2%, and 97.2%, respectively. However, when compared to manual calculations, the CNN tended to underestimate both TLV (0.43 cm^3^ versus 0.398 cm^3^, *p* < 0.005) and TLU (32.89 versus 40.93, *p* = 0.049). Kaplan–Meier analysis demonstrated that the TLV and TLU calculated automatically by the CNN significantly correlated with patient overall survival (both *p* < 0.005).

### 3.8. Assessing Treatment Response based on PSMA PET Scans

Acar et al. [33] developed and compared a few different methods of ML to differentiate active bone metastasis from post-treatment (chemotherapy, ADT, radiotherapy, or 177LU-psma) sclerotic bone lesions in PCa patients. Data being input into the ML includes hounsfield unit (HU), histogram data, shape-based data, and second-order textural analysis data. Acar et al. demonstrated that the weighted KNN ML algorithm had the highest accuracy (73.5%) and area under the curve (76%) to differentiate sclerotic lesions from metastasis with 73.5% sensitivity and 73.7% specificity.

Duriseti et al. [34] assessed castration-sensitive PCa (csPCa) patients who underwent ADT with or without local intervention such as radiotherapy or surgery. These patients underwent PSMA PET-CT before treatment and three months or more after treatment. aPROMISE was employed to automatically calculate a PSMA score which considers lesion volume and SUV. The baseline median PSMA score for each anatomical site was as follows: prostatic bed (21.6), lymph nodes (5.3), bone (2.2), and composite (9.7). The median PSMA score for all anatomical areas decreased to zero post-treatment. The decrement in median PSA post-treatment was 100% (range: 68–100%). There was a significant association between the change in PSMA score and post-treatment PSA, which led to their postulation that PSMA score measured by aPROMISE post-treatment can be used to quantify treatment response.

Moazemi et al. [26] developed an ML trained on pre-treatment PSMA PET-CT radionics and clinical parameters of metastatic PCa patients planned for 177Lu-PSMA. He found that radiomics features (PET_Min, PET_Correlation, CT_Min, CT_Busyness and CT_Coarseness) and clinical parameters (ALP1 and Gleason score) showed best correlations with changes in PSA level post-treatment. The ML algorithm could predict response to 177Lu-PSMA treatment with 80% AUC, 75% sensitivity, and 75% specificity.

## 4. Discussion

This systematic review comprehensively analyses the current state of AI’s ability to assess mPCa with or without lymph node involvement. All the included studies were published within the last four years underscoring the growing interest in incorporating AI into the assessment of medical imaging.

Previous studies have demonstrated variations in the performance and reporting of PSMA PET-CT [11]. Standardization of radiological reporting is crucial to ensure that the results are reproducible, consistent, and comprehensible [36]. Standardized reporting will facilitate the interpretation of data in both clinical and research settings. Efforts to standardize PSMA PET reporting have been promising, but labour and time-intensive [12]. The current systematic review demonstrated that AI could help standardize the reporting of PSMA-PET CT as guided by the PROMISE criteria and maintain low inter-reader variability [24] whilst reducing the workload by automation of organ segmentation [27] and anatomy allocation [30]. In future studies, it will be interesting to have a head-to-head comparison of PSMA PET reporting with and without AI to evaluate the following outcomes: inter-reader variability, intra-reader-variability, learning curve for a nuclear medicine trainee, time needed to complete a PSMA PET report, changes in capacity and workload with the use of AI, and analysis of the influence of AI on nuclear medicine decision to elucidate any bias AI may introduce in the final reporting.

This systematic review also demonstrated the relatively high sensitivity (between 62 and 97%) and accuracy (AUC up to 98%) of AI’s ability to detect all types of metastatic disease [30,31,32]. Although it may outperform nuclear medicine physicians in some instances, it does carry a low and widely variable PPV (between 39.2 and 66.8%) [29]. This supports the idea that AI tools are just an adjunct and not meant to replace nuclear medicine physicians. Perhaps these tools should not be utilised by trainees for formal reporting, but by experienced nuclear medicine physicians who can proofread these AI-generated reports. These AI tools may still be used as educational tools during practice to help trainees with their detection of positive sites. An additional benefit of such a utilisation model is that corrections from an experienced nuclear medicine physician will further improve the diagnostic capability of AI algorithms through internal feedback mechanisms [37]. It is unclear why there is a large variation in outcomes between included papers. Possible explanations could stem from variations in developmental data (for example favoring low-volume metastasis), usage of different PSMA tracers, or variations in scan acquisitions. The decision-making process of an AI algorithm is often not transparent and has been described as a black box [38]. Until AI models become more interpretable and explainable, the lack of transparency may present a barrier to its integration into clinical practice. Long-term follow-up studies are also needed to understand if this improved detection of metastatic disease translates into changes in long-term oncological outcomes. An area that has not been explored by the included papers is whether AI could accurately quantify the number of hotspots.

The study by Johnsson et al. [27] demonstrated that aPROMISE was able to identify suspicious regional lymph nodes with a 91.5% sensitivity. The ability to distinguish malignant from benign lymph nodes is important in the decision-making of PCa treatment. Especially given the current landscape where we are widely adopting PSMA PET as the pre-treatment staging modality. Due to the limitation of time, our existing literature guiding treatment is from the conventional staging (CT AP and WBBS) era. It is a dilemma as to how to manage patients with positive regional lymph nodes on PSMA PET-CT that were negative on conventional staging. In this instance, AI could bridge the gap between evolving imaging technologies and treatment strategies. If the AI algorithm can non-invasively determine that a regional lymph node has a very low likelihood of being malignant on PSMA PET, the patients be able to avoid the morbidity associated with lymph node dissection or extended field radiation. Similarly, if the AI algorithm can determine that a non-regional lymph node that was PSMA avid has a very low likelihood of malignancy, the patient may still benefit from active treatment (prostatectomy or radiotherapy). Further research into this area is required. One of the limitations of PSMA PET is its false negative rate of 12% and false positive rate of 3% when evaluating pelvic lymph node metastases [39]. The lower detection rate appears to be related to the smaller metastatic lymph node, with up to 91% of undetected metastatic lymph nodes being less than 5 mm. It will be interesting if future studies can evaluate AI’s ability to improve the detection of these small lymph nodes.

We appreciate there are limitations to the included studies. Firstly, many of the studies were retrospective, with small sample sizes, and did not describe the demographic or clinicopathological information of the included patients. Exclusion criteria were also not mentioned in most of the included studies. Separate data sets for validation and training were not used in some studies. Additionally, the only study which compared different types of ML models was by Moazemi et al. [26]. Therefore, no recommendations can be made at this stage regarding which subtype of AI algorithm is optimal for evaluating mPCa on PSMA PET-CT. Lastly, the heterogeneity of included patients and outcomes precluded a meta-analysis.

We should be cautious of the limitations and shortcomings of AI, particularly in clinical use. The AI models are only as good as the data it is trained on, if the data set is not representative of a diverse patient population, a bias may arise [40]. Additionally, AI models are often trained on controlled data sets which may not be representative of real-life scenarios where there are variations in scan equipment, acquisition time of scans, and type of PSMA tracer given [41,42]. Lastly, biopsy is the current gold standard for confirmation of metastatic disease, however, performing multiple biopsies in patients with high-volume metastatic disease is not feasible. Therefore, the ground truth used in the development of these AI models is limited by visual diagnosis by experienced nuclear medicine physicians. Therefore, results and analysis (such as sensitivity and accuracy) are dependent on the diagnostic accuracy of the nuclear medicine physician to differentiate between benign versus metastatic lesions on the PSMA PET scans.

As we head towards an era of personalised medicine, future studies could consider combining radiomics with clinicopathological factors (such as PSA or Gleason score) in their AI algorithms to see if it improves diagnosis. Two of the included studies assessed the capability of AI to evaluate metastatic disease and predict its response to treatment; however, more studies are needed before we can determine whether AI could influence treatment decisions. Future studies should also differentiate between low and high-volume mPCa during the development of AI models as they may present differently radiologically with a worse disease state having higher PSMA avidity [43,44] Another area where AI could be implemented in the future is to to improve image acquisition and processing quality [45]. Ongoing studies also present interesting applications of AI on PSMA PET scans such as guiding theranostics [46,47].

## 5. Conclusions

AI can detect lymph node involvement and metastatic disease with high accuracy (area under the curve of 98%) and sensitivity (between 62 and 97%). Additional benefits of AI include differentiating metastatic bone lesions from post-treatment bony sclerosis, estimating tumour burden for prognostic purposes, predicting treatment response of mPCa, automating time-consuming tasks with high accuracy (such as organ segmentation and anatomical allocation of lesions), and reducing inter-reader variability during reporting. Although the preliminary findings appear promising, larger prospective studies with reproducible results are needed before AI can be considered for assimilation into clinical practice.

## Figures and Tables

**Table 1:** Characteristics and results of included studies.

Author and Year	Study Objective	AI Model and Study Design	PSMA Tracer Used	Inclusion Criteria	Sample Size of (Training/Validation/Test)	Data Input	Comparator	Algorithm Performance	Strength	Limitations
Nickols et al., 2021 [24]	Evaluate aPROMISE’s ability to reduce inter-reader variability of PSMA PET-CT	DL Multi-centre Retrospective	^18^F- PSMA PET-CT	veterans with intermediate- or high-risk PCa who underwent PSMA scan	NR/NU/109 No cross-validation	NR	Between two nuclear medicine physicians	Cohen pairwise k-agreement for PCa staging between two readers was high (0.82 for miN0M0, 0.90 for miN1M0, 0.77 for miN0M1b.)	Moderate sample size Using external data to evaluate an existing DL software	Retrospective Selective study population (only veterans)
Johnsson et al., 2022 [27]	Based on aPROMISE, evaluate the sensitivity of automated detection of potential lesions	DL Multi-centre Retrospective	^18^F-PSMA PET-CT	1. high-risk PCa planned for RP with PLND) 2. radiologic evidence of recurrent or metastatic PCa and considered feasible for biopsy	NR/235/295 No cross-validation	PSMA PET-CT scans annotated by experienced nuclear medicine readers for location, SUVmax, SUVpeak, SUVmean, and uptake volume.	NR	Sensitivity of detecting lesion with metastasis: 91.5% for regional lymph node 90.6% for all lymph node 86.7% for bone	Large sample size Using external data to evaluate an existing DL software	Retrospective Demographic and clinicopathological characteristics of included patients were not reported. No cross-validation
Leung et al., 2022 [28]	Develop an ML to perform classification of PSMA uptake and correlate to PSMA-RADS	DL Multi-centre Retrospective	^18^F-PSMA PET-CT	Patients who underwent ^18^F-PSMA PET-CT	267 patients had 3794 lesions divided into: 2302/760/732	Scans were segmented by four nuclear medicine physicians then CNN extracted radiomic features and tissue-type information	Probability score compared against PSMA-RADS categories on a t-SNE scatter plot	PSMA-RADS classification at lesion level AUROC 0.87 and accuracy of 0.52. Patient level AUROC 0.9 and accuracy of 0.77. Probability score of PSMA-RADS-1 and 2 was 0.19, PSMA-RADS-3 was 0.5, PSMA-RADS-4 and 5 was 0.86	Large sample size Has both training and validation set	Retrospective Demographic and clinicopathological characteristics of included patients were not reported. Demographic and clinicopathological characteristics of included patients were not reported.
Trägårdh et al., 2023 [29]	Develop and validate a CNN for detecting and quantifying tumour burden (TLV and TLU) of lymph node metastases and bone metastases	CNN Single-centre Retrospective	^18^F-PSMA PET-CT	initial staging of high-risk prostate cancer or for the detection of sites of suspected recurrent disease.	420/120/120 No cross-validation	One independent nuclear medicine physician segmented and annotated the scan. Three main inputs include CT image, PET image, and multi-channel organ mask	Sensitivity of nuclear medicine physicians for detecting lymph nodes (78%) and bone metastasis (59%)	Sensitivity of CNN for detecting lymph nodes (79%) and bone metastasis (62%) correlations of TLV and TLU between CNN and nuclear medicine physicians were all statistically significant and ranged from R = 0.53 to R = 0.83.	Large sample size Data set for testing separate from training data Compared to several nuclear medicine physicians	Single center The same data set is for training and validation. Demographic and clinicopathological characteristics of included patients were not reported.
Capobianco et al., 2021 [30]	Develop and evaluate CNN to classify PSMA uptake into anatomical location and determine if it is suspicious for cancer	CNN Single-centre Retrospective	^68^Ga-PSMA PET-CT	1. Primary staging of PCa or for assessment of BCR 2. PSMA-ligand PET-CT for all other indications of PCa.	121/NU/52 4-fold cross-validation	Nuclear medicine physician labelled PSMA uptake into anatomical location and suspicion for PCa. Data from ^18^F-FDG PET-CT scans was added to determine if improved CNN	Compared to nuclear medicine physician assessment	CNN had an average precision of 80.4% [CI: 71.1–87.8] for suspicious uptake identification, 77% (CI: 70.0–83.4) accuracy for anatomical classification of suspicious findings, agreement for identification of regional lymph node involvement (81%) and metastatic stage (77%)	Demonstrated combining training information from ^18^F-FDG PET/CT and ^68^Ga-PSMA-11 PET/CT led to improved accuracy	Single center Small data set for testing and no separate data set for validation Demographic and clinicopathological characteristics of included patients were not reported.
Erle et al., 2021 [31]	Comparing and validating ML algorithms in classifying pathological uptake in PCa	ML Single-centre Retrospective	^68^Ga-PSMA PET-CT	PCa patients who underwent PSMA PET-CT for either staging or treatment control	72/NU/15 3-fold cross-validation	77 radiomics features calculated using InterView FUSION software from 2452 manually delineated hotspots on PSMA PET-CT	Testing with a hold-out set of 15 patients	AUC = 98% Sensitivity = 97% Specificity = 82%	A detailed explanation of radiomics features used in the development	Small sample size No histopathological confirmation of metastasis
Moazemi et al., 2020 [32]	Develop and evaluate ML algorithm in differentiating non-specific from malignant PSMA uptake	ML Single-centre Retrospective	^68^Ga-PSMA PET-CT	Follow-up staging or consideration of radionuclide therapy for PCa patients who previously underwent treatment (active or systemic treatment)	48/24/NU 5-fold cross-validation	40 textural features calculated using InterView FUSION software from 2419 hotspots determined by nuclear medicine physicians on PSMA PET-CT	Compared to nuclear medicine physician assessment	AUC = 98% Sensitivity = 94% Specificity = 89%	A detailed explanation of radiomics features used in the development Developed and compared five different ML algorithms	Small sample size Patients underwent various treatments (hormonal versus chemotherapy versus radiotherapy)
Kendrick et al., 2022 [25]	Develop and evaluate a CNN to extract prognostic biomarkers (TLV and TLU) from PSMA PET-CT	CNN Single-centre Prospective	^68^Ga-PSMA PET-CT	BCR PCa following active treatment who received PSMA PET-CT before further surgery, radiotherapy, or systemic treatment. follow up scans 6 months later	262 */NU/75 * 53 negative scans used as control 5-fold cross-validation	Lesions for each patient scan were manually delineated by an expert Nuclear Medicine Physician	Testing with a hold-out set of 75 patients.	Accuracy = 94.5% Sensitivity = 93.3% Specificity = 96.2% TLV and TLU from CNN were associated with overall survival (both *p* < 0.005)	Large sample size Prospective Used negative scans as a control	Single center
Acar et al., 2019 [33]	Develop ML to differentiate PCa bony metastatic versus sclerotic (responded to treatment) on PSMA PET-CT	ML Single-centre Retrospective	^68^Ga-PSMA PET-CT	PCa with known bone metastasis and who were previously treated	75/NU/NU 10-fold cross-validation	Lesion marked by nuclear medicine physician on LifeX software analysis which extracted HU, 5 histogram data, 3 shape-based data, and 32 s-order textural analysis data	Results from cross-validation	AUC = 76% Accuracy = 73.5% Sensitivity = 73.5% Specificity = 73.7% Weighted KNN ML algorithm could differentiate metastasis bony from completely responded lesions	Used completely responded sclerotic lesions as control	Retrospective Small sample size
Duriseti et al., 2023 [34]	Quantifying treatment response by correlating changes in aPROMISE PSMA score to PSA changes	CNN Site NR Retrospective	^18^F-PSMA PET-CT	csPCa who underwent PSMA PET-CT before and 3 months or more after surgery, radiotherapy, and/or ADT	NR/NU/30 No cross-validation	aPROMISE was used to identify, quantify, and calculate changes in PSMA tracer avid disease	Compared to post-treatment PSMA PET-CT	Baseline prostate bed PSMA scores were correlated with baseline PSA (*p* < 0.001). Nodal (*p* = 0.53) and bony (*p* = 0.65) baseline PSMA scores did not correlate with baseline PSA. Changes in PSMA scores were significantly correlated with corresponding decreases in PSA for composite and nodal disease, but not for prostate bed or bony disease	Clinicopathological characteristics of included patients reported.	Small sample size No separate data set for development and testing
Moazemi et al., 2021 [26]	Develop ML to predict response to 177Lu-PSMA treatment using Baseline PSMA-PET-CT scans and clinical parameters	ML Single-centre Retrospective	^68^Ga-PSMA PET-CT	Advanced PCa scheduled for treatment with 177Lu-PSMA	56/27/NU 3-fold cross-validation	14 clinical parameters And 73 radiomics features were calculated using InterView FUSION software from a 2070 hotspot determined by a nuclear medicine physician on PSMA PET-CT	a permutation test (null hypothesis = permuted distribution of ground truth labels could have resulted in similar prediction scores)	AUC = 80% Sensitivity = 75% Specificity = 75% Radiomics features (PET_Min, PET_Correlation, CT_Min, CT_Busyness and CT_Coarseness) and clinical parameters such as Alp1 and Gleason score showed best correlations with change in PSA	Included clinical parameters in the development of the AI model A detailed explanation of radiomics features used in the development	Small sample size Single center

Acronyms: automated Prostate Molecular Imaging Standardized Evaluation (aPROMISE), Prostate-specific Membrane Antigen Reporting and Data System (PSMA-RADS), Convolutional neural network (CNN), Deep learning (DL), Prostate cancer (PCa), Castration-sensitive prostate cancer (csPCa), Biochemical recurrence (BCR), Radical prostatectomy (RP), Pelvic lymph node dissection (PLND), Androgen deprivation therapy (ADT), Chemotherapy or ADT (systemic therapy), Confidence interval (CI), T-distributed stochastic neighbour embedding (t-SNE) Hounsfield unit (HU), Total lesion volume (TLV), Total lesion uptake (TLU), Area under the curves (AUCs), Area under the receiver operating characteristic curve (AUROC), Positive predictive value (PPV), NR (not reported), NU (not used) * indications for ePLND in Cysouw et al. study were either an ≥8% risk score of LNI based on the Memorial Sloan Kettering Cancer (MSKCC) nomogram or any high-risk feature (≥T3, Gleason > 7, PSA > 20 ng/mL).

**Table 2:** The prediction model risk of bias assessment tool (PROBAST) of included studies.

Study	ROB				Applicability Concerns			Overall	
	Participants	Predictors	Outcome	Analysis	Participants	Predictors	Outcome	ROB	Applicability
Nickols et al., 2021 [24]	Low	Low	Low	Low	Low	Unclear	Unclear	Low	Unclear
Johnsson et al., 2022 [27]	Low	Low	Low	Low	Low	Low	Low	Low	Low
Leung et al., 2022 [28]	Unclear	Low	Low	Low	Unclear	Low	Low	Low	Low
Trägårdh et al., 2023 [29]	Low	Low	Low	Low	Low	Low	Low	Low	Low
Capobianco et al., 2021 [30]	Low	Low	Low	Low	High	Unclear	Unclear	Low	High
Erle et al., 2021 [31]	Low	Low	Low	Low	Low	Low	Low	Low	Low
Moazemi et al., 2020 [32]	Low	Low	Low	Low	Low	Low	Low	Low	Low
Kendrick et al., 2022 [25]	Low	Low	Low	Low	Low	Low	Low	Low	Low
Acar et al., 2019 [33]	Low	Low	Unclear	High	Low	High	Low	High	High
Duriseti et al., 2023 [34]	Unclear	Low	Low	Low	Unclear	Unclear	Unclear	Low	Unclear
Moazemi et al., 2021 [26]	Low	Low	Low	Low	Low	Low	Low	Low	Low

ROB = Risk of bias.

## Supplementary Materials

The following supporting information can be downloaded at: https://www.mdpi.com/article/10.3390/cancers16030486/s1. Reference [48] is cited in the Supplementary Materials.
